# Impact of foreign direct investment on income inequality: Evidence from selected Asian economies

**DOI:** 10.1371/journal.pone.0281870

**Published:** 2023-02-15

**Authors:** Maksudjan Yuldashev, Ulugbek Khalikov, Fazliddin Nasriddinov, Nilufar Ismailova, Zebo Kuldasheva, Maaz Ahmad

**Affiliations:** 1 Department of Finance and Business analysis, Tashkent State University of Economics, Tashkent, Uzbekistan; 2 Department of Corporate Governance, Tashkent State University of Economics, Tashkent, Uzbekistan; 3 Department of World Economy, Tashkent State University of Economics, Tashkent, Uzbekistan; Southeast University, BANGLADESH

## Abstract

The United Nations lists 17 Sustainable Development Goals for Agenda 2030, one of which is SDG-10, which focuses on eradicating inequality and addressing critical regional and global challenges. The fight against income inequality is heavily dependent on foreign direct investment all over the world. In this connection, the present study aimed to investigate the individual and interactive impact of foreign direct investment, human capital, and economic growth on income inequality by employing the interactive model. Based on the panel data set covering ten counties spanning each region of Asia from 1990 to 2020. In light of the slope homogeneity, cross-sectional dependency tests, and Westerlund co-integration test, we discover that all of the variables are cointegrated over the long run. A cross-sectional IPS (CIPS) unit root test is employed to check stationarity. Additionally, the study used the Augmented Mean Group (AMG) approach to produce accurate results in estimation. The results confirm that FDI affects inequality negatively. However, the impact of FDI is more effective in the presence of human capital. It means that human capital deepens the effect of FDI on inequality; the country will be more effective in reducing inequality by having a higher level of human capital and consider it a more powerful tool to bring equality. To reduce inequality, it is suggested that a policy mix of FDI and HC could be made.

## 1. Introduction

Inequality is one of the most common problems in developing countries, even in developed countries. During the last several decades, policymakers have focused on economic growth and income inequality. It is not only a measure of unequal income distribution, but it also has societal implications, such as on public policies, economic development, and institutional quality. It is the imbalance in income distribution between rich and poor. The rich got more, while the poor were mostly deprived. According to the World Inequality Report (2022) [1], Currently, the top 10 percent of the world’s population receives 52 percent of the global income, while the bottom 50 percent own only 8.4 percent of it. This means that most of the people on earth are quite poor. Nearly 4 billion people on earth survive on less than 6.70 dollars per day [2]. Experts in Global Economic Inequality (2018) also report that low-income people can expect their income to rise by around 4,000 dollars until 2035.The statistics showed that the world’s economy grew by about 14 percent during the last five years. Conversely, the world’s Foreign Direct Investment (FDI) flows rise by approximately 23 percent. This flow was 1.35 trillion dollars in 2000, and it is expected to increase to 1.58 trillion dollars by 2021. Similarly, human capital is vital for attracting FDI flows and sophisticated technologies [3]. It is believed that investing in human capital will raise productivity and spur economic development. According to an estimation, about 62 percent of the world’s population is developed with human capital and active in economic activities. The Global Human Capital Index (2019) makes evident that the world’s 25 countries recently improved their human capital by about 70%, while the rest remained stagnant. Asia’s economic growth and income inequality are snowballing [4]. Asia is considered the fastest-rising economic zone in the world. According to the International Monetary Fund annual report (IMF, 2022), the nominal GDP of Asia was 41.78 trillion US dollars in 2022. In recent times, income inequality has been one of the most profound challenges for Asian countries. The majority of Asia’s developing countries have seen an increase in inequality. Therefore, the income gap between the poor and rich is the key concern for policy designers in Asian countries (Asian Development Bank, 2019). There are 800 million people in Asia who are still surviving below 1.25 dollars per day, and 1.7 billion out of 4.46 billion people live below 2 dollars per day. FDI is considered the major contributor to the pace of growth in many economies. It also creates employment opportunities and causes technological diffusion, which may help developing countries boost their growth. According to statistics by UNCTAD (2022), Asia is the largest FDI recipient region in the world. It attracted about 40 percent of the world’s FDI in 2022. In this context, during 2020–21, 16 percent of FDI increased in East Asia, 44 percent rose in South-East Asia, 12 percent increased in Central Asia, and 59 percent of the increases were found in West Asia [5]. According to the Global Human Capital Index (2017), 93 percent of the world population of developed countries contributes more than 95 percent of the world’s GDP. It indicates that human capital plays an essential role in the economic growth of any country. It is observed that the level of Government Consumption Expenditure (GCE) increased, leading to an increase in human capital of about 52 percent from 1990 to 2019 in East Asia and South Asia. This increase aimed to boost employment and social protection, education, skills, and health. The above aspects are responsible for providing services and deliberately supporting national economies. In short, these statistics show that Asia is facing the problem of income inequality, this region is the most FDI-attracting region in the world, and that there has been a very little increase in human capital compared to developed countries.

Therefore, the aim of this study is many folds, firstly, we investigate the impact of FDI, FDI*HC, and FDI*GDP on income inequality in three models for ten Asian developing countries from 1990 to 2020. Secondly, we selected a panel of 10 developing countries from Asia, which two countries are selected from each region, i.e., Turkey and Iran from Western Asia, Indonesia and the Philippines from South-East Asia, India and Pakistan from South Asia, China and South Korea from Eastern Asia, and Kazakhstan and Uzbekistan from Central Asia. Thirdly, for empirical analysis, we used three separate models. The first model deals with the individual variables, and the second and third models deal with the interactive terms. The study’s novelty lies in using interactive models to differentiate between marginal and average impacts of individuals and interactive terms. Finally, this study used the most recent techniques of [6] for slope heterogeneity and CIPS for stationarity checks. Furthermore, this study used Westerlund co-integration and AMG techniques to examine the long-run relationship between the variables under consideration. At the end of the study, panel causality is tested using the Pairwise Dumitrescu Hurlin causality test.

It is found in the study that an increase in the inflows of FDI has caused a reduction in income inequality; moreover, as much as human capital and economic growth increased, inequality decreased by a massive amount in the study area. The distributional effects of FDI, HC, and GDP more effective at reducing inequality. These findings suggested that if policymakers want to improve the quality of education and health care, they should work harder to reduce inequalities. Education and health care can improve labor force efficiency and contribution efficiency. It not only reduces inequality but also positively affects other aspects of the economy.

The remaining paper is set up in such a way that Section 2 consists of a literature review. The methodology is discussed in Section 3, while results and discussions are made in Section 4. In the last section, conclusions and policy recommendations are framed.

## 2. Literature review

The significance of income inequality and economic growth is a hot debated issue in the current literature and has been comprehensively examined and overawed by [4,7–9]. Nemours studies are conducted to elucidate different factors that determine income inequality. These factors are economic, social, demographic, and political, i.e., economic growth, tax rate, inflation, education, unemployment, population growth, institutional reforms, law and order situations, etc. In recent literature, FDI and inequality are framed in the same picture and have gained remarkable popularity. In this connection [10,11], disclose that FDI contributes significantly to determining inequality. Conversely, the studies conducted by [12,13] argued that FDI did not take part in the variation of inequality. The literature reports mixed findings; therefore, there is a need for a comprehensive study to get a clear picture of the association between FDI and income inequality.

Similarly, in past literature, the researcher presented several variables that examined the association between income inequality and FDI [10,14–16]. However, the study by [8] used the variables of inflation, unemployment, private investment, and human capital to determine inequality [10]. came up with a new financial development variable and a new idea for a financial development term that works together with FDI. They found a negative impact of FDI on income inequality, while financial development deteriorates the effect of FDI on inequality. Financial development accelerates the rate of inequality. Similarly [17], examined the effects of globalization and human capital on income inequality. [18] examined the causal relationship between income inequality and poverty. Similarly [19], added a new variable of international trade, and its impact on income inequality. The most recent study of [20] raises the question, "Does globalization affect income inequality?" Moreover, they used new variables, i.e., human capital, remittance inflows, urbanization, and value-added to services and industry sectors. However, the role of interactive terms (FDI*HC and FDI*GDP), which show the average effects of interaction on income inequality through variations in the level of human capital and economic growth, is totally ignored. It is generally accepted that an increase in FDI leads to enhanced income inequality [21–24] while studies undertaken by [25] are of the view that FDI deteriorates inequality. So, policymakers are still trying to find a precise and stable way to link the variables so that FDI can have the most positive effect on the economy as a whole.

Numerous techniques have been used in literature to get their objectives related to income, different aspects of human lives, and inequality, such that [26] employed the Shapley value decomposition approach and GMM for the economy of China. [7,27] have used cross-country OLS and GMM estimators for a panel of 54, 113, and 26 countries, respectively. Another technique of 2SLS is employed by [28] for China. Non-linear ARDL, a comparatively recent approach, is used by [24,29] has employed several techniques to examine the desired objectives. These techniques are Fixed Effect, 2SLS, and GMM. In the last, study of [19] applied pooled OLS, LSDVC, and system GMM, estimator. Literature has observed different techniques to achieve their objectives, but these techniques have some limitations. The previous studies employed LM and Harris-Tzavalis panel unit root tests. The prior one is suitable in the presence of slop homogeneity but not efficiently applicable in the case of cross-sectional dependency. The latter doesn’t deal with cross-sectional dependency or slope homogeneity. Another unit root test is also employed, which is the CSADF test. This test deals with cross-sectional dependency but does not compatible with slope heterogeneity. The conventional unit root tests described above are inefficient in terms of CS-dependency. Therefore, we have used the cross-sectional IPS panel unit-root test presented by [30], which has high compatibility with slop heterogeneity and CS-dependency. The techniques used for parameter estimates have some severe limitations too. These limitations are in turn as, FMOLS techniques are generally used for small sample sizes with 1st-difference I(1) of stationarity. Similarly, the Johansen Panel co-integration technique applies in the case of 1st order of stationarity. GMM estimators provide efficient results for a panel with a large N and a small T. Furthermore, many other first-generation co-integration techniques are also employed in past studies, which provide dubious results due to the residual value of cross-sectional dependency and slop heterogeneity. Therefore, we employ second-generation panel co-integration techniques introduced by [31], which provide efficient output in the presence of CS-dependency and slope heterogeneity in both the long and short runs. Moreover, the causal relationship is also examined with the help of the Dumitrescu–Hurlin panel causality test.

It is obvious from the literature that various studies have been conducted regarding income inequality with economic growth and other socio-economic factors. These factors include foreign direct investment, population growth, inflation, unemployment, human capital, tax rate, etc. These relationships provided marginal impacts, while the average and distributional effects were ignored in the literature. In this context, this study contributed to the literature by including the concept of interactive terms in our models, i.e., FDI*HC and FDI*GDP, to examine the distributional impact of FDI and economic growth on income inequality. Moreover, this is the first study framed for Asian countries in which interactive terms are used. Another attractive point of the study lies in the selection of the countries in Asia. Equal weight has been given to each region. The exact numbers of the countries are drawn from each region of Asia, i.e. Turkey, Iran, Indonesia, the Philippines, India, Pakistan, China, South Korea, Kazakhstan, and Uzbekistan.

## 3. Methodology

### 3.1 Model specification

To examine the impact of FDI on income inequality in the presence of human capital and economic growth in Asian countries. This study used other essential variables from the literature, i.e., GDP and GFCE. Ten (10) countries are selected from different regions of Asia (every two countries from a region depending on their importance related to inequality and data availability). For the same purpose, the following model is used:

$${GINI}_{it}=f({FDI}_{it},{HC}_{it},{GDP}_{it},{GFCE}_{it})$$

$${lnGINI}_{it}=\beta_{0}+\beta_{1}{lnFDI}_{it}+\beta_{2}{lnHC}_{it}+\beta_{3}{lnGDP}_{it}+\beta_{4}{lnGFCE}_{it}+\beta_{5}{lnFDI*HC}_{it}+\mu_{it}$$ (1)

Where GINI represents income inequality, HC is human capital, GDP indicates economic growth, FDI shows foreign direct investment, and GFCE is the combination of all general govt final consumption expenditure. This study has used four different variables that closely affect income inequality i.e. FDI, HC, GDP and GFCE and interactive terms of FDI*HC which leads to Eq (2):

$${lnGINI}_{it}=\beta_{0}+\beta_{1}{lnFDI}_{it}+\beta_{2}{lnHC}_{it}+\beta_{3}{lnGDP}_{it}+\beta_{4}{lnGFCE}_{it}+\beta_{5}{lnFDI*HC}_{it}+\mu_{it}$$ (2)

And the interactive term of FDI*GDP reveals Eq (3):

$${lnGINI}_{it}=\beta_{0}+\beta_{1}{lnFDI}_{it}+\beta_{2}{lnHC}_{it}+\beta_{3}{lnGDP}_{it}+\beta_{4}{lnFDI*GDP}_{it}+\mu_{it}$$ (3)

In the above three (03) equations, the term ’i’ represents cross-sections i.e., countries and ‘t’ represents time span. Whereas β_0_ represents the intercept term, β_1_, β_2_, β_3_ and β_4_ show the parameters of the independent variables and ‘μ’ is the disturbance term. The signs of the variables are expected to be different depending on different aspects of the economies i.e. economics, politics and society. The literature reported that the sign of the impact of FDI on income inequality is negative means increase in FDI leads to reduce inequality which can be presented as FDI affecting inequality $\frac{{\partial GINI}_{it}}{\partial {FDI}_{it}}$ < 0. It is supposed that FDI negatively affects income inequality. Human Capital affects income inequality inversely i.e. $\frac{{\partial GINI}_{it}}{\partial {HC}_{it}}$ < 0, and the same is expected from GDP and GFCE i.e. $\frac{{\partial GINI}_{it}}{\partial {GDP}_{it}}$ < 0 and $\frac{{\partial GINI}_{it}}{\partial {GFCE}_{it}}$ < 0. The interactive term effects are also expected negatively and more impressive to reduce income inequality in the selected countries from Asia i.e. $\frac{{\partial GINI}_{it}}{\partial {FDI*HC}_{it}}$ < 0 and $\frac{{\partial GINI}_{it}}{\partial {GDP*HC}_{it}}$ < 0 with a high magnitude. It indicates that the expected inverse impact of FDI inflow and GP growth in the presence of HC is supposed to be more effective i.e. $\frac{{\partial GINI}_{it}}{\partial {FDI*HC}_{it}}$ ˃ $\frac{{\partial GINI}_{it}}{\partial {FDI}_{it}},\frac{{\partial GINI}_{it}}{\partial {HC}_{it}}$ and $\frac{{\partial GINI}_{it}}{\partial {GDP*HC}_{it}}$ ˃ $\frac{{\partial GINI}_{it}}{\partial {GDP}_{it}},\frac{{\partial GINI}_{it}}{\partial {HC}_{it}}$.

### 3.2 Estimation techniques

The first step in the examination, heterogeneity of slopes, and cross-sectional dependency of the variables, are taken into account at the start of the analysis. For cross-sectional dependency, we use the most commonly used test in the literature, presented by [30]. To check the stationarity of the panel data, we used the second-generation unit root test, i.e., cross-sectional IPS, and the long-run relationship was tested with the newest co-integration technique, named Westerlund co-integration technique. This technique assumed no cross-sectional dependency among the sections and no heterogeneity between the slops. Homogeneity in slops and cross-sectional dependency are of the utmost importance and can be presented in turn. The equation for homogeneity in slop is as follows:

$${\tilde{\Delta }}_{SH}={\left(N\right)}^{1/2}{\left(2K\right)}^{1/2}\{1/N(\tilde{S})-k\}$$ (4)

$${\tilde{\Delta }}_{ASH}={\left(N\right)}^{1/2}{\left[2K(T-k-1)/(T+1)\right]}^{1/2}$$ (5)

In Eqs (4) and (5), Delta tilde and adjusted delta tiled show ${\tilde{\Delta }}_{SH}$ and ${\tilde{\Delta }}_{ASH}$ respectively.

The stationarity of the variables is tested with the help of a panel unit root test known as cross-sectional IPS (CIPS). It is a second-generation panel unit root test with the property of dealing with cross-sectional dependency. The equation defines it as:

$$\hat{CIPS}=\frac{1}{N}\sum_{i-1}^{N}{CADF}_{i}$$ (6)

To examine the existence of the long-run relationship among the variables of the models in individual and interactive form as well, we employed the Westerlund cointegration technique proposed in 2007 for ten Asian economies. It is based on the null hypothesis that there is no co-integrational relationship between the variables included in the model. The rejection of the null hypothesis will lead to the existence of a long-run relationship. The definitional equation of the Westerlund test is:

$$\alpha_{t}(L)\Delta Y_{it}=\Gamma_{1it}+\Gamma_{1it}+\beta_{i}(Y_{it}-1-{\tilde{\alpha }}_{i}X_{it-1})+\varphi_{i}(L)v_{it}+\mu_{it}$$ (7)

Where,

$\delta_{1i}=\beta_{i}(1)\vartheta_{2i}-\beta_{i}\varphi_{1i}+\beta_{i}\vartheta_{2i}$ and, $\Gamma_{2i}=-\beta_{i}\varphi_{2i}$

The description of the α_i_ and β_i_ is the in the above equations are the co-integrational vector between the variables included in the model and estimated coefficient of the error term respectively. The followings are the test statistics:

$$G_{t}=N^{-1}\sum_{i-1}^{N}[{\dot{\alpha }}_{i}/SE({\dot{\alpha }}_{i})]$$ (7.1)

$$G_{a}=N^{-1}\sum_{i-1}^{N}[T{\dot{\alpha }}_{i}/{\dot{\alpha }}_{i}(1)]$$ (7.2)

$$P_{T}={\dot{\alpha }}_{}/SE({\dot{\alpha }}_{})$$ (7.3)

$$P_{a}=T\dot{\alpha }$$ (7.4)

In Eqs 9.1, 9.2, 9.3 and 9.4, the group and panel statistics are represented by G_a_, G_t_, P_a_, and P_t_. To get the value of the error correction parameter ‘ά’ by rewriting the following equation:

$$P_{a}=T\dot{\alpha }$$

$$\dot{\alpha }=P_{\alpha }/T$$

$$\overset{´}{\alpha }=\frac{P_{\alpha }}{T}$$

This shows the speed of adjustment towards the equilibrium in the short run.

The presence of cross-sectional dependency affects the efficiency of estimators estimated with general estimation techniques, i.e., panel regression, co-integration, etc. As the data variables are found to be cross-sectionally dependent, this study used an AMG estimator to estimate the coefficients of variables in the models. AMG is proposed by [32,33] to test the robustness of the coefficients in the model and the model as a whole. The technique has preference over the other methods because of its applicability in the presence of cross-sectional dependence and heterogeneity. This technique can be defined as:

$$y_{it}=\overset{´}{\alpha_{i}}+\beta_{1i}x_{it}+\varphi_{i}h(\bar{\varphi }f_{t})+\mu_{it}$$ (8)

In the above equation, ‘f_t’_ is the unobserved /unseen/unnoticed common factor that permits a heterogeneous effect on the explained variable.

The Harris-Tzavalis, LM, and cross-sectional ADF panel unit root tests were not used in this study because the Harries-Tzavalis panel unit root test only applies when there is cross-sectional dependency and heterogeneity. As with the LM panel unit root test, it deals with heterogeneity but not cross-sectional dependency. In the same way, cross-sectional ADF deals with cross-sectional dependency but not with heterogeneity. As a result, we used the CIPS panel unit root test to determine the stationarity of the panel data in this study; it is also appropriate in the case of a small sample size.

Literature suggests that in the presence of the CD and heterogeneity, we cannot use conventional panel co-integration techniques, because they do not provide efficient results [34]. Therefore, this study used Westerlund’s (2007) co-integration technique, which provides efficient results even when error terms are dependent cross-sectionally and have no common factor restriction. The most recent studies by [13] have used the same technique. For parameters, estimation techniques like Augmented Mean Groups (AMG) and Common Correlated Effects Mean Group (CCEMG) work best because they deal with and heterogeneity in panel data analysis.

### 3.3 Data description

The core objective of the present study is to examine the distributional impact of FDI, human capital, economic growth, government final consumption expenditure on income inequality in selected Asian countries. We use the annual data for 10 selected less developed Asian countries from 1990 to 2020, however, the description of the variables are given below in Table 1.

**Table 1:** Descriptions of the variables and sources of the data.

Variables	Descriptions	Sources
Gini index	Range from zero to a hundred, the index near to zero shows high equality, while near to a hundred shows high unequal distribution of income.	SWIID
FDI	The stock of FDI as a percentage of GDP.	WDI
HC	Human capital index, based on years of schooling and returns to education.	PWT
GDP	GDP per capita	WDI
GFCE	Government Final Consumption Expenditure as a percentage of GDP	WDI
FDI*HC	An interaction term between FDI and human capital, to explain the marginal impact of FDI on inequality.	Calculated by Authors
FDI*GDP	An interactive term between FDI and GDP per capita	Calculated by Authors

## 4. Empirical results

This study investigates the average and distributional impacts of FDI and economic growth with other explanatory variables on income inequality for selected ten less-developed Asian countries from 1990 to 2020. The cross-sectional dependency test result is shown in Table 2 that all variables are statistically significant and cross-sectionally dependent except the variable government final consumption expenditure, which is insignificant. The null hypothesis is rejected at 1 percent for each variable of cross-section dependency. Furthermore, the slope homogeneity tests confirmed the acceptance of the alternative hypothesis, making the results suspicious of the model under consideration. In the second part of Table 2, a slope homogeneity test is employed to investigate the status of slope homogeneity or heterogeneity. The null hypothesis for the test is that there are no homogeneous slopes, while the slopes are heterogeneous in the alternative hypothesis. It is clear from the P-values, i.e., less than 0.01, which confirms the rejection of the null hypothesis. Thus, it is concluded that slopes are homogenous.

**Table 2:** Cross-sectional dependence and slope homogeneity tests.

Cross-sectional Dependence Test		
**Variables**	**CD test**	**P-Value**
LnGINI	2.40**	0.016
LnFDI	24.33***	0.000
LnHC	23.06***	0.000
LnGFCE	0.40.6	0.685
LnGDP	31.91***	0.000
lnFDI*HC	29.95***	0.000
lnFDI*GDP	31.64***	0.000
**Slope homogeneity test**		
**Model-I**	Value	P-values
Delta_tilde	20.61***	0.000
Adj Delta_tilde **Model-II** Delta_tilde Adj Delta tilde **Model-III** Delta_tilde Adj Delta tiled	23.14*** 17.45*** 20.045*** 17.46*** 20.048***	0.000 0.000 0.000 0.000 0.000

Note

*** denotes 1% significance level.

This study employs the CIPS unit root test, introduced by Pesaran (2007). The key objective of this technique is to check the stationarity between all variables included in all three models. CIPS is a second-generation unit root technique specially constructed for cross-sectional and panel dependencies. The CIPS results are very important because they also take into account the cross-sectional and panel dependencies suggested by individual common factors. From the Table 3 confirms the existence of mixed orders of stationarity. lnFDI and lnGFCE are stationary at level with 1% significance, whereas lnGINI, lnHC, lnGDP, lnFDI*HC, and FDI*GDP are stationary in first-difference of order with 1% significance. Therefore, the mixed order of stationarity between the variables recommended the Westerlund co-integration technique and the AMG estimator for estimation.

**Table 3:** CIPS unit root test.

**Variable**	**Level**		1^st^ Difference			
	Intercept & Trend		Intercept & Trend			Order
**lnGINI**	-0.873		-2.829**			I(1)
**lnFDI**	-2.889**		-4.478***			I(0)
**lnHC**	-1.908		-3.876***			I(1)
**lnGFCE**	-2.981**		-4.456***			I(0)
**lnGDP**	-1.458		-4.035**			I(1)
lnFDI*HC	-1.072		-4.387***			I(1)
lnFDI*GDP	-0.983		-4.019***			I(1)

Note

***,**, * represent 1%, 5%, 10% level of significance, respectively.

This study analyzed Westerlund’s (2007) co-integration technique to check the long-run equilibrium between income inequality with other independent variables of economic growth, FDI, human capital, government consumption, and especially the distributional effect of interaction term i.e. FDI*HC and FDI*GDP. Westerlund’s (2007) panel co-integration technique results are shown in Table 4. Group mean and panel mean is denoted by G_t_, G_a_, P_t_, and P_a,_ respectively. The analyses confirm a stable long-run association between our interested variables. Hence, to find the error correction parameter (ά), we have to put the value of $P_{a}=T\overset{´}{\alpha }$. For that reason, we get the parameter value of $\overset{´}{\alpha }=\frac{P_{\alpha }}{T}=\frac{-9.82}{10}$ = -0.982 for the simple model, while $\frac{-9.77}{10}$ = -0.977 for both Interactive Model-I and Interactive Model-II. These outcomes are evident that all statistics are significantly based on P-values thus, the null hypothesis is rejected. Therefore, we are convinced to assume the presence of co-integration between the variable under-examined.

**Table 4:** Westerlund co-integration test.

Models	G_t_	G_a_	P_t_	P_a_
**Simple Model** lnGINI = ƒ(lnFDI, lnHC, lnGFCE, lnGDP)	-8.130***	-16.715***	-7.127**	-9.823**
**Interactive Model-I** lnGINI = ƒ(lnFDI, lnHC, lnGFCE, lnGDP, lnFDI*HC)	-8.364***	-16.609***	-6.679**	-9.774*
**Interactive Model-II** lnGINI = ƒ(lnFDI, lnHC, lnGFCE, lnGDP, lnFDI*GDP)	-8.364***	-17.60***	-6.679**	-9.774**

Note: ***, **, * represent 1%, 5%, 10% level of significance, respectively.

To check the robustness of the model, we use the AMG estimator. The results of the AMG test are shown in Table 5 for the simple model, which describes that FDI and human capital are negatively related to income inequality. At the same time, government final consumption expenditure and economic growth are positively associated. It reveals that all variables included in the model have a significant role in determining income inequality. The results of the AMG estimator state that the co-efficient value of FDI is statistically significant and negative for income inequality in all three models. It was observed that a 1% increase in FDI inflows decreased income inequality by 0.071%, while the impact of FDI in Interactive Model-I is 0.037% and is 0.05% in Interactive Model-II. The same impact is also reported by [10,16,25,35]. Conversely, the study conducted by [10] experienced a positive association between them. He believed that FDI caused a rise in income inequality because of poor economic governance and inefficient institutional reforms. Additionally, the coefficient of human capital is negative and statistically significant for income inequality. This study elucidated a long-run relationship between human capital and income inequality in selected Asian developing economies. It is founded that a 1% increase in human capital contributes to decreasing income inequality by 0.044% in the simple model, 0.048% in the interactive Model-I, and 0.049% in interactive Model-II. The negative impact between human capital and income inequality is also reported by [36–38] in their studies. However, the recent research by [39] reported quite the opposite relationship, i.e., that an increase in human capital leads to increased income inequality. Moreover, the coefficient of economic growth is positive and significant for income inequality. It indicates that a 1% increase in economic growth contributes to an increase in the unequal distribution of income by 0.021% in the simple model, while 0.039% in the interactive Model-I, and 0.027% in the interactive Model-II. The present study’s findings are parallel with the outcomes of the studies conducted by [40,41]. They contend that the uppermost wealthy class receives a greater share of the growth. On the other hand, some studies contradict the findings of our study; one of them is conducted by [42]. The impact of government final consumption expenditure in developing Asia is undesirable because it accelerates the face of unequal distribution of income. The result indicated that a 1% increase in government consumption can spur income inequality by 0.097%, 0.092%, and 0.090% in the simple Model, interactive Model-I, and interactive Model-II, respectively. [43] also reported the same impact in his most recent study. The government expenditure will increase income inequality, but later on, it will reduce the disparity. Finally, the coefficient values for both interactive terms (FDI*HC and FDI*GDP) are estimated, and both are negatively and significantly related to income inequality. The novelty of the study lies in interactive terms. The interactive terms coefficient values are 0.141% and 0.152%, respectively. It means that a 1% increase in the interactive terms, i.e., FDI*HC, leads to a decrease in income inequality by 0.141% and FDI*GDP by 0.152%.

**Table 5:** AMG estimator.

Variables	Simple Model	Interactive Model-I	Interactive Model-II
**lnFDI**	-0.071** (0.0142)	-0.037*** (0.004)	-0.050** (0.013)
**lnHC**	-0.044** (0.010)	-0.048** (0.010)	-0.049** (0.014)
**lnGDP**	0.020*** (0.004)	0.039*** (0.010)	0.027*** (0.0058)
**lnGFCE**	0.097** (0.014)	0.0923** (0.018)	0.090** (0.019)
**lnFDI** * **HC** **lnFDI** * **GDP**	---- ----	-0.141** (0.016) ----	---- -0.152** (0.017)
**Wald Test**	4.502***	6.641***	7.092***

Note: ***, **, * represent 1%, 5%, 10% level of significance, respectively.

It is clear from the above three models that the impact of individual terms, i.e., FDI and HC, is negative and significant. However, the distributional impact of FDI and human capital is more effective. FDI can even contribute to a reduction in income inequality, but an increase in human capital deepens the impact of FDI on income inequality. On the other hand, the impact of growth on income inequality in the simple model is positive, but the impact of growth changed with FDI in interactive terms Model-II. We can further say that the impact of FDI in the presence of human capital and growth is more effective in reducing income inequality, which justifies the theme of the study.

In Table 6, the result of Dumitrescu-Hurlin’s (2012) panel causality test is reported. It shows a bi-directional causal relationship between inequality and FDI. Additionally, human capital and income inequality also have a bi-directional causal relationship. The current result is supported by [44]. We found a two-way causal relationship between inequality and GDP; the present result is in line with the findings of the study conducted by [45]. We also found a bi-directional relationship between government final consumption expenditure and income inequality. On the other hand, a unidirectional causal association is found between the interactive variable (FDI*HC) i.e. run from interactive term to income inequality. A unidirectional relationship is also reported between interactive term (FDI*GDP) and income inequality, running from interactive term to income inequality.

**Table 6:** Pairwise dumitrescu-hurlin panel causality test.

**Direction of causality**	**W-Stat.**	**Z.bar-Stat.**	**Prob.**
**FDI → GINI**	5.328***	4.067***	0.000
**GINI → FDI**	3.666**	1.907**	0.056
**GINI → HC**	7.355***	6.703***	0.000
**GDP → GINI**	5.923***	4.841***	0.000
**GINI → GDP**	4.307***	2.740***	0.006
**GFCE → GINI**	8.729***	5.688***	0.000
**GINI → GFCE**	5.318***	4.054***	0.000
FDI*HC → GINI	5.702***	4.553***	0.000
GINI → FDI*HC	3.232	1.342	0.179
FDI*GDP → GINI	6.065***	5.025***	0.000
GINI → FDI*GDP	3.533*	1.734*	0.082

Note: ***, **, * represent 1%, 5%, 10% level of significance, respectively.

Initially, the cross-sectional dependency test confirmed that all variables are cross-sectionally dependent. Therefore, we employed the slope homogeneity test and rejected the null hypothesis of slope homogeneity. The cross-sectional IPS unit root test showed the mixed order of integration in variables, suggesting to use the AMG estimator and Westerlund co-integration techniques. This technique found stability in the long-run relationship between the variables under consideration. Results reported that the individual term is less effective while the interactive terms are more effective in reducing income inequality in selected Asian developing economies.

## 5. Conclusion

This study aims to find the impact of FDI on income inequality in the context of human capital in Asian developing countries. Ten developing countries are selected for this study based on FDI status, inequality, human capital, and data availability regarding countries from 1990 to 2019. Initially, this study tested cross-sectional dependency among the variables under consideration, revealing the models cross-sectional dependency. A slope homogeneity test is also employed and confirms the presence of slope homogeneity in the model. For stationarity, the CIPS unit-root test is used to check the order of stationarity of the variables. A mixed order of stationarity is revealed. This study used the AMG technique to get the long-run elasticity of estimates. FDI and human capital are negative and statistically significant, while the impact of GDP and GFCE is positively related to income inequality. In the other models consisting of interactive terms, it was observed that the impact of interactive terms was more effective than that of individual variables. It is concluded that FDI is more effective in reducing income inequality in the context of human capital and GDP. Later, the pairwise Dumitrescu-Hurlin panel causality test is employed to know the causal relationship among the model’s variables.

### 5.1 Policy recommendation

Income inequality is one of the most serious economic problems that afflicts many people in society. Different policies have been devised to tackle it. In this connection, internal and external policies are also put into practice. In internal policies, many tax policy reforms have also been successfully framed in the past, and beneficial outcomes have been achieved through them. By sending money from rich regions to poor regions, we can close the gaps between them. Moreover, fiscal policies can safeguard equal access to education and augment human capital, which can help the poor reduce income inequality. Other economic factors are considered responsible for it and can reduce it. External capital inflows like remittances, FDI and foreign aid, are considered the sources of the reduction in income inequality. But the present study tried to examine the effect of FDI inflow to developing countries on income inequality even more effectively in the presence of human capital.

This study suggests that special attention be paid to FDI policies to attract foreign investors and reduce income inequality in developing Asia. FDI provides an opportunity for the labor force to work in MNCs, leading to rising employment opportunities. Along with external policies, the governments are also advised to give options to their nationals to boost human capital by increasing the level and standard of education further to deepen the impact of FDI on income inequality. Human capital can play the role of a catalyst to increase the labor force’s productivity, which is the theme of the endogenous growth theory. The productivity of the labor force will increase FDI even more by making investors more money. This study believes that external and internal policy coordination would reduce income inequality in developing countries more efficiently.

### 5.2 Limitation of the study

A drawback to this study is that we do not consider all Asian countries, so in future researcher should make a panel of the whole Asian economies to extend this study. Furthermore, it is suggested that if we use Human Resource Development (HRD) instead of human capital, represented by the secondary school enrolment rate, it may further improve the level of inequality because HRD is a more broad term as compared to human capital. Hence, in future research, it can be used in studies that could be more effective and suitable. We also do not investigate whether the FDI and governance interactions matter for reducing income inequality. We leave these topics for future study.
